# Childhood maltreatment, prefrontal-paralimbic gray matter volume, and substance use in young adults and interactions with risk for bipolar disorder

**DOI:** 10.1038/s41598-020-80407-w

**Published:** 2021-01-08

**Authors:** Dylan E. Kirsch, Valeria Tretyak, Sepeadeh Radpour, Wade A. Weber, Charles B. Nemeroff, Kim Fromme, Stephen M. Strakowski, Elizabeth T. C. Lippard

**Affiliations:** 1 Department of Psychiatry and Behavioral Sciences, Dell Medical School, University of Texas at Austin, 1601 Trinity Street, Stop Z0600, Health Discovery Building, Austin, TX 78712 USA; 2 Waggoner Center for Alcohol and Addiction Research, University of Texas, Austin, TX USA; 3 Institute for Neuroscience, University of Texas, Austin, TX USA; 4 Department of Psychology, University of Texas, Austin, TX USA; 5 Institute of Early Life Adversity Research, University of Texas, Austin, TX USA

**Keywords:** Bipolar disorder, Stress and resilience, Risk factors

## Abstract

Childhood maltreatment is associated with adverse effects on the brain, and an increased risk for psychopathology, including mood and substance use disorders. Individuals vary on the degree to which they exhibit neurobiological and clinical differences following maltreatment. Individuals with bipolar disorder exhibit greater magnitude of maltreatment-related prefrontal-paralimbic gray matter volume (GMV) deficits compared to typically developing individuals. It is unclear if greater structural differences stem from greater neural vulnerability to maltreatment in bipolar disorder, or if they relate to presence of other clinical features associated with childhood maltreatment, e.g., elevated prevalence of comorbid substance use disorders. To investigate this, we compared young adults with a family history of bipolar disorder (n = 21), but who did not fulfill diagnostic criteria for bipolar disorder, with typically developing young adults without a family history of bipolar disorder (n = 26). Participants completed structural neuroimaging, clinical and family history interviews, and assessment of childhood maltreatment and recent alcohol and cannabis use patterns. We examined relations between childhood maltreatment and prefrontal-paralimbic GMV by modeling main effects of maltreatment and family history group by maltreatment interactions on prefrontal-paralimbic GMV. We also examined relations between maltreatment and associated GMV changes with recent alcohol and cannabis use. Childhood maltreatment correlated with lower ventral, rostral and dorsolateral prefrontal and insular cortical GMV across all participants regardless of the presence or absence of familial history of bipolar disorder. However, exploratory analyses did reveal greater maltreatment-related GMV differences in individuals with prodromal symptoms of depression. Lower insula GMV was associated with greater frequency of cannabis use across all participants and greater quantity of alcohol use only in those with familial risk for bipolar disorder. Results suggest familial risk for bipolar disorder, and presumably genetic risk, may relate to outcomes following childhood maltreatment and should be considered in prevention/early intervention strategies.

## Introduction

Early life stress (ELS), including childhood maltreatment, increases risk for a wide range of major psychiatric disorders, including bipolar disorder^1,2^. High rates of ELS are reported in individuals with both bipolar disorder, with estimates of ELS prevalence as high as 57%^3^. ELS has been related to neural changes that likely increase risk for bipolar disorder. Structural brain abnormalities in prefrontal-paralimbic systems observed following ELS are similar to structural abnormalities reported in bipolar disorder^4–6^. As individuals vary to the degree to which they exhibit neurobiological differences associated with ELS^6^, it is critical to identify factors that may contribute to variability in these neurobiological differences, and associated clinical outcomes.

Studies have suggested greater structural abnormalities associated with ELS in individuals with bipolar disorder, compared to healthy adults^7,8^, including greater magnitude of gray matter volume (GMV) differences in prefrontal-paralimbic systems (e.g., ventral prefrontal and insular cortices^9^). Greater structural changes exhibited in prefrontal-paralimbic regions following ELS in bipolar disorder may be related to prevalence and severity of ELS observed in this population^3^ or to other disease-related factors that may alter prefrontal-paralimbic structure (e.g., medication exposure^10^ or disease-related GMV loss^11–13^). As prior work has suggested ELS interacts with family history of unipolar depression to influence brain structure^14^, findings could converge to suggest greater vulnerability of prefrontal-paralimbic systems to ELS in bipolar disorder, perhaps stemming from genetic factors that contribute to risk for bipolar disorder. No study has addressed whether ELS interacts with family history of bipolar disorder (and hence presumed genetic vulnerability) to impact prefrontal-paralimbic structures.

ELS also increases risk for substance use disorders (SUDs), including alcohol use disorders (AUDs)^15^, and ELS is associated with higher prevalence of comorbid SUDs in bipolar disorder, compared to prevalence of SUDs in healthy adults^16^. Substance use has been suggested to be an intermediate step between ELS and bipolar disorder^15^, and greater GMV differences following ELS in bipolar disorder may relate to high rates of SUDs in this population^17,18^. Genes that confer risk for bipolar disorder may also increase risk for SUDs^19^, however, it is unknown if familial risk for bipolar disorder interacts with ELS and associated brain changes to contribute to substance use during young adulthood, further structural brain changes following ELS, and ultimately risk for the development of bipolar disorder, SUDs, and their comorbidity.

The current study sought to determine if ELS interacts with familial risk for bipolar disorder to contribute to differences in neural structure of prefrontal-paralimbic systems, and if ELS and related neural differences relate to current substance use patterns in young adults. We hypothesized an interaction between ELS, specifically childhood maltreatment, and familial risk for bipolar disorder on prefrontal-paralimibic GMV. Additionally, we hypothesized childhood maltreatment and maltreatment-related GMV differences would be associated with substance use, with the familial risk subgroup showing a stronger association. We recruited young adults with first-degree family member(s) diagnosed with bipolar disorder, but who themselves did not fulfill diagnostic criteria for bipolar disorder (FH+), and a group of typically developing young adults without a first-degree family member diagnosed with bipolar disorder (FH−). Participants completed clinical and family history interviews, including assessment of childhood maltreatment and recent alcohol and cannabis use, and structural magnetic resonance imaging (MRI) scans. In this preliminary study, ELS-related differences in GMV within prefrontal-paralimbic a priori regions of interest (ROIs) were modeled across all participants, and interactions with familial risk for bipolar disorder were investigated. ROIs included the insular and ventral, dorsal, and rostral prefrontal cortices. Lower GMV in these regions have been reported following childhood maltreatment^6^ and found to distinguish individuals with bipolar disorder who prospectively develop alcohol and cannabis use problems^18^. Based on our hypothesis, we predicted childhood maltreatment would inversely correlate with GMV in prefrontal-paralimbic regions, with greater GMV differences in the FH+, compared to in the FH− group. We also predicted childhood maltreatment and maltreatment-related prefrontal-paralimbic GMV differences would show an inverse relation with past month alcohol and cannabis use, with greater childhood maltreatment and greater maltreatment-related reductions in GMV associated with greater substance use in the FH+, compared to the FH− group. As individuals in the FH+ group presumably vary in their inherited risk for bipolar disorder, we also conducted an exploratory analysis aiming to determine if differences in maltreatment-related GMV exist within the FH+ group. Specifically, we investigated differences between FH+ individuals with prodromal depression history, compared to FH+ individuals without prodromal depression history, because higher polygenic risk for bipolar disorder is associated with unipolar depression in youth with familial risk for bipolar disorder^20^.

## Methods and materials

### Participants

Participants included 47 young adults (21 FH+, 26 FH−). Table 1 details demographic, and familial characteristics stratified by group (FH+, FH−). Exclusion criteria included a diagnosis of bipolar disorder, major medical or neurological illness, including head injury with loss of consciousness for ≥ 5 min, an IQ < 85, or other MRI contraindication. Participants with familial risk for bipolar disorder were not excluded if they had a history of unipolar depression or an anxiety disorder because depression and anxiety are considered prodromal symptoms of, and risk factors for, both bipolar disorder and SUDs^21,22^. Additionally, across all participants, a history of SUDs was not an exclusion factor to facilitate recruiting a generalizable sample^23^ and to explore relations among maltreatment, related prefrontal-paralimbic system GMV, and recent alcohol and cannabis use patterns. As familial risk for bipolar disorder and other psychopathology (e.g. familial risk for SUDs) often coincide, FH− participants were not excluded for a family history of non-bipolar disorder psychiatric conditions (e.g. depression, anxiety, ADHD, and alcohol/substance us problems) to help account for additional familial psychopathology risk factors present in the FH+ group^24^, and to facilitate recruiting a FH− group with greater similarity in prevalence of childhood maltreatment to the FH+ group.

**Table 1:** Demographic, childhood maltreatment, and familial characteristics stratified by group.

	FH− (N = 26)	FH+ (N = 21)	p value	FH+DEP− (N = 9)	FH+DEP+ (N = 12)	p value
**Demographics**						
Mean age (SD)	21 (2)	21 (2)	0.7	21 (2)	21 (2)	0.9
Number of females (%)	19 (73)	17 (81)	0.7 ^F^	6 (67)	11 (92)	0.3^F^
Mean WASI-II FSIQ-2^a^ (SD)	118 (14)	117 (12)	0.7	117 (14)	117 (11)	1
**Childhood Maltreatment (CTQ)**^**b**^						
CTQ total score (SD)	33 (8)	45 (15)	** < 0.001**^**U**^	36 (8)	51 (16)	**0.01**
CTQ range	25 – 54	27 – 85		27 – 53	27–85	
**Comorbidities**^**c**^						
Current alcohol use disorders (AUDs):						
AUD—mild (%)	0	1 (5)	0.5^F^	0	1 (8)	1^F^
Past alcohol use disorders (AUDs):						
AUD—mild (%)	1 (4)	2 (10)	0.6^F^	1 (11)	1 (8)	1^F^
Current substance use disorder (SUDs):						
Cannabis use disorder—mild (%)	1 (4)	1 (5)	1^F^	1 (11)	0	0.4^F^
Cannabis use disorder—moderate (%)	1 (4)	0	1^F^	0	0	–
Past substance use disorder (SUDs):						
Cannabis use disorder—mild (%)	1 (4)	0	1^F^	0	0	–
Cannabis use disorder—severe (%)	0	1 (5)	0.5^F^	0	1 (8)	1^F^
Past major depressive episode (%)	0	12 (57)	** < 0.001**^**F**^	0	12 (100)	** < 0.001**^**F**^
Anxiety disorders^d^ (%)	0	7 (33)	**0.002**^**F**^	2 (22)	5 (42)	0.6^F^
**Urinary toxicology screen**						
Tetrahydrocannabinol (%)	3 (12)	1 (5)	0.3^F^	1 (11)	0	0.4^F^
Amphetamines (%)	1 (4)^e^	0	1^F^	0	0	–
**Family history**						
Bipolar disorder (%)	0	21 (100)	** < 0.001**^**F**^	9 (100)	12 (100)	1^F^
Depression (%)	9 (35)	11 (52)	0.3	4 (44)	7 (58)	0.7^F^
Anxiety (%)	5 (19)	12 (57)	**0.01**	5 (56)	7 (58)	1^F^
ADHD (%)	2 (8)	3 (14)	0.6^F^	1 (11)	2 (17)	1^F^
Alcohol use problems (%)	7 (27)	18 (86)	** < 0.001**^**F**^	7 (78)	11 (92)	0.6^F^
Substance use problems (%)	3 (12)	12 (57)	** < 0.001**^**F**^	4 (44)	8 (67)	0.4^F^

Entire sample [bipolar disorder family history negative (FH−) vs. bipolar disorder family history positive (FH+)] and FH+ subgroup [FH+ individuals without a history of unipolar depression (FH+DEP) vs. FH+ individuals with a history of unipolar depression (FH+ DEP+)] between-group differences in age, IQ, and CTQ were compared using a two-sample t-test. All other factors were examined with a Mann–Whitney Wilcoxon Test, Chi-square, or Fisher Exact tests, as appropriate.

^a^FSIQ-2 represents the composite score for the full-scale intelligence quotient comprising verbal comprehension and matrix reasoning subtests on the Wechsler Abbreviated Scale of Intelligence-Second Edition (WASI-II).

^b^Childhood Trauma Questionnaire.

^c^No individuals presented with comorbid AUD and SUD. ^d^Anxiety disorders included generalized anxiety disorder, specific phobia, panic disorder, and social anxiety disorder.

^e^Individual who tested positive for amphetamines also tested positive for tetrahydrocannabinol.

^U^Represents p-values calculated with Mann–Whitney Wilcoxon Test.

^F^Represents p-values calculated with Fisher exact test.

Current and lifetime psychiatric diagnoses and clinical characteristics were obtained using the Structured Clinical Interview for DSM-V-Research Version^25^*.* Verbal comprehension and matrix reasoning subtests of the Wechsler Abbreviated Scale of Intelligence-Second Edition (WASI-II;^26^) were used as a measure of intelligence quotient (IQ; FSIQ-2). Family history of bipolar disorder, depression, anxiety, attention deficit hyperactivity disorder (ADHD), alcohol and substance use disorders was assessed using the Family History—Research Diagnostic Criteria—Epidemiological Version^27^. All study procedures were approved by the University of Texas at Austin Institutional Review Board, and written informed consent was obtained from all participants. All methods were performance in accordance with the relevant guidelines and regulations. Participants were instructed to abstain from alcohol and drug use for 24 h before their scan. On the day of the scan, urine screens were administered to test for pregnancy and substances of abuse. Four participants (1 FH+, 3 FH−) had positive drug tests (see Table 1 for urine toxicology results).

### Assessment of childhood maltreatment and alcohol/cannabis use

#### Childhood maltreatment

All individuals completed the Childhood Trauma Questionnaire (CTQ), a 28-item retrospective self-report test measuring five subtypes of maltreatment (emotional abuse and neglect, physical abuse and neglect, and sexual abuse)^28^. A total CTQ score was obtained by summing the five maltreatment subtype scores. Total CTQ score was used as a continuous measure based on prior research suggesting childhood trauma exhibits a dose-dependent association between multiple types of trauma types on both neuroimaging findings and clinical outcomes^29–31^. Table 1 details childhood maltreatment characteristics stratified by groups (FH+, FH−).

#### Alcohol/cannabis use

The Timeline Follow Back was used to obtain daily reports of alcohol and cannabis use for four weeks prior to the date of MRI assessment^32,33^. Using a calendar marked with holidays and specific days, participants reported which days they consumed alcohol and cannabis and the number of standard drinks they consumed on these days. Recent alcohol use was quantified by determining maximum number of drinking days per week and average number of drinks per drinking day over the prior four weeks. Recent cannabis use was quantified by determining maximum number of cannabis use days per week over the prior four weeks. Table 2 details recent alcohol and cannabis use stratified by group. Participants were also asked at what age they initiated alcohol use (i.e. age of first drink, not just a sip from an adult’s glass, and not including drinking as part of religious ceremonies).

**Table 2:** Past month alcohol and cannabis use.

	FH− (N = 25)	FH+ (N = 17)	p value	FH+DEP− (N = 7)	FH+DEP+ (N = 10)	p value
**Recent alcohol use**						
Maximum number of drinking days/week (SD)	1.8 (1.5)	1.8 (1.8)	0.8	2.2 (0.9)	1.4 (1.2)	0.4
Average number of drinks/drinking day (SD)	2.3 (2.4)	2.1 (1.7)	0.8	1.5 (1.5)	2.5 (1.8)	0.7

	FH− (N = 6)	FH+ (N = 7)	p value	FH+DEP− (N = 2)	FH+DEP+ (N = 5)	p value
**Recent cannabis use**						
Maximum number of cannabis use days/week (SD)	2 (1.7)	2.4 (1.4)	0.6	3.5 (0.7)	2 (0.6)	0.3

Past month alcohol use [mean and standard deviation (SD)] stratified by group [bipolar disorder family history negative (FH−) vs. bipolar disorder family history positive (FH+) individuals who completed the Timeline Follow Back and reported lifetime alcohol use] and FH+ subgroups (FH+ individuals without a history of unipolar depression (FH+DEP−) vs. FH+ individuals with a history of unipolar depression (FH+DEP+) who completed the Timeline Follow Back and reported lifetime alcohol use]. Past month cannabis use stratified by group in individuals who completed the Timeline Follow Back and reported past month cannabis use. Between group differences were calculated using Mann–Whitney Wilcoxon Tests.

### Structural neuroimaging: magnetic resonance imaging acquisition and preprocessing

All imaging was performed with a single 3-T Siemens Skyra MR scanner using a 32-channel head coil located at the University of Texas at Austin Biomedical Imaging Center (UT BIC). Sagittal structural MRI images were acquired with a three-dimensional MPRAGE T1-weighted sequence with parameters: repetition time (TR) = 1900 ms, echo time (TE) = 2.42 ms, matrix = 224 × 224, field of view = 220 × 220mm^2^, 192 one-mm slices without gap and one average. All scans were assessed visually for movement and noise artifacts. Statistical Parametric Mapping-12 (SPM12, http://www.fil.ion.ucl.ac.uk/spm) was used to pre-process structural data with the DARTEL toolbox in SPM12 as previously described^18^.

### Statistical analysis

#### Between-group differences in demographic, childhood maltreatment, familial characteristics, and alcohol/cannabis use patterns

Shapiro-Wilke tests were used to assess normality of data. *T*-tests (two-tailed) or Mann–Whitney–Wilcoxon tests were performed to assess between-group differences (FH+ vs. FH−) in age, IQ, CTQ total score, and recent alcohol and cannabis use patterns, as appropriate. Chi-square or Fisher’s exact tests were used to assess between-group differences in categorical variables, including sex (female/male), self-history of AUDs and SUDs, past major depressive episode, anxiety disorder(s), urine toxicology screens, and family history of unipolar depression, anxiety disorder(s), ADHD, and alcohol and substance use problems, as appropriate.

#### Neuroimaging data analysis: CTQ relations to prefrontal-paralimbic gray matter volume

For primary hypothesis testing, SPM12 was used to model the relation between total CTQ score and prefrontal-paralimbic GMV in a priori hypothesized ROIs (insular and ventral, dorsal, and rostral prefrontal cortices) across all participants, with group (FH+, FH−) and sex as covariates. Sex was included as a covariate due to established sex-differences in the variables of interest^18,34^. Findings within a priori ROIs were considered significant at p < 0.005 (uncorrected) and clusters ≥ 20 voxels. This threshold was chosen to balance type I and type II errors in preliminary studies as previously described^18,35^. Group (FH+, FH−) by CTQ interactions on GMV, covarying sex, were modeled in SPM12. Mean GMV from clusters showing a significant relation with total CTQ across all participants and from clusters showing significant group by CTQ interactions was calculated and extracted. For clusters showing a significant group by CTQ interaction, relations between CTQ total score and extracted GMV were modeled stratified by group, while covarying sex, to investigate group differences driving a significant interaction. Studies have shown greater severity of childhood maltreatment is associated with more severe neural outcomes^34^. Therefore, following a significant interaction, a sensitivity analysis was conducted with extracted GMV to assess group by CTQ interactions after removing four FH+ individuals with the highest CTQ scores and four FH− with lowest CTQ scores so that groups (FH+ vs. FH−) did not differ in terms of average CTQ score and CTQ score range would be similar between groups.

#### Neuroimaging data analysis: childhood maltreatment and associated prefrontal-paralimbic gray matter volume relations with alcohol/cannabis use patterns

Alcohol and cannabis use measures were not normally distributed (Shapiro Wilke test, p < 0.05); therefore, a logarithmic transformation was applied to these measures. Across all participants who completed the Timeline Follow Back and reported lifetime alcohol use (N = 42; 17 FH+, 25 FH−), relations between total CTQ with recent alcohol use were investigated, with sex, group (FH+, FH−), and age of alcohol initiation included as covariates. Group (FH+ vs. FH−) by total CTQ interactions, covarying sex and age of alcohol initiation, were also modeled with alcohol use measures as the dependent variables. Age of alcohol initiation was included as a covariate because childhood maltreatment is associated with earlier age of alcohol initiation^36^, earlier initiation is associated with greater alcohol use^37–39^, and earlier initiation is associated with family history of problematic alcohol and other substance use^40^, which is commonly observed in individuals with familial risk for bipolar disorder. Following a significant interaction, relations between total CTQ and alcohol use were modeled within each group, covarying sex and age of alcohol initiation, to investigate group differences driving a significant interaction. The relation between total CTQ and recent cannabis use was explored in individuals who reported past month cannabis use, with sex and group included as covariates. Analysis was not conducted across the entire sample and interactions with group were not investigated for cannabis use owing to sample size [only 13 (28%) of participants reported smoking cannabis over the past month (FH+: N = 7; FH−: N = 6)]. Parallel models were conducted, as described above, except with extracted GMV (from regions that showed a significant association with maltreatment) as the independent variable and alcohol and cannabis use as the dependent variables. Results were considered significant at p ≤ 0.05 uncorrected for these planned analyses. All significant findings are reported below.

#### Exploratory analyses: FH+ subgroups

In an exploratory analysis, the FH+ group was stratified into two subgroups: FH+ with a history of depression (FH+DEP+, N = 12) and FH+ without a history of depression (FH+DEP−, N = 9) to explore effects of childhood maltreatment in a presumably higher genetic risk group. History of depression was defined as having met criteria for a past major depressive episode on the Structured Clinical Interview for DSM-V-Research Version. Between group differences in demographics, childhood maltreatment, familial characteristics, and alcohol/cannabis use patterns were investigated, as described above. Table 1 details demographic, childhood maltreatment, and familial characteristics stratified by family history subgroup (FH+DEP+; FH+DEP−). Table 2 details recent alcohol and cannabis use stratified by family history subgroup. FH+ subgroup (FH+DEP+, FH+DEP−) by CTQ interactions on GMV, covarying sex, were modeled in SPM12, as described above. Mean GMV from clusters showing a significant group (FH+DEP+ vs. FH+DEP−) by CTQ interactions was calculated and extracted. Relations between CTQ total score and extracted GMV were modeled stratified by group, while covarying sex, to investigate group differences driving a significant interaction. Also as described above, following a significant interaction, a sensitivity analysis was conducted with extracted GMV to assess group by CTQ interactions after removing four FH+DEP+ individuals with the highest CTQ scores so that groups (FH+DEP+ vs. FH+DEP−) did not differ in terms of average CTQ score and CTQ score range would be similar between groups (see Supplemental Table 1 for comparison of matched FH+ subgroups).

## Results

### Between-group differences in demographic, childhood maltreatment, and familial characteristics, and alcohol/cannabis use patterns

CTQ total scores were higher in the FH+ group, compared to the FH− group (p < 0.001). The FH+, compared to the FH− group, had more individuals with a personal past history of unipolar depression (p < 0.001) and an anxiety disorder (p = 0.002), and higher prevalence of family history of an anxiety disorder (p = 0.01) and alcohol and substance use problems (both: p < 0.001). See Table 1 for information regarding differences in participant demographics, childhood trauma, and family history characteristics. Groups did not differ in recent alcohol or cannabis use. See Table 2 for information regarding differences in participant alcohol and cannabis use patterns.

### Neuroimaging analysis

#### CTQ relations to prefrontal-paralimbic gray matter volume

**Figure 1:**
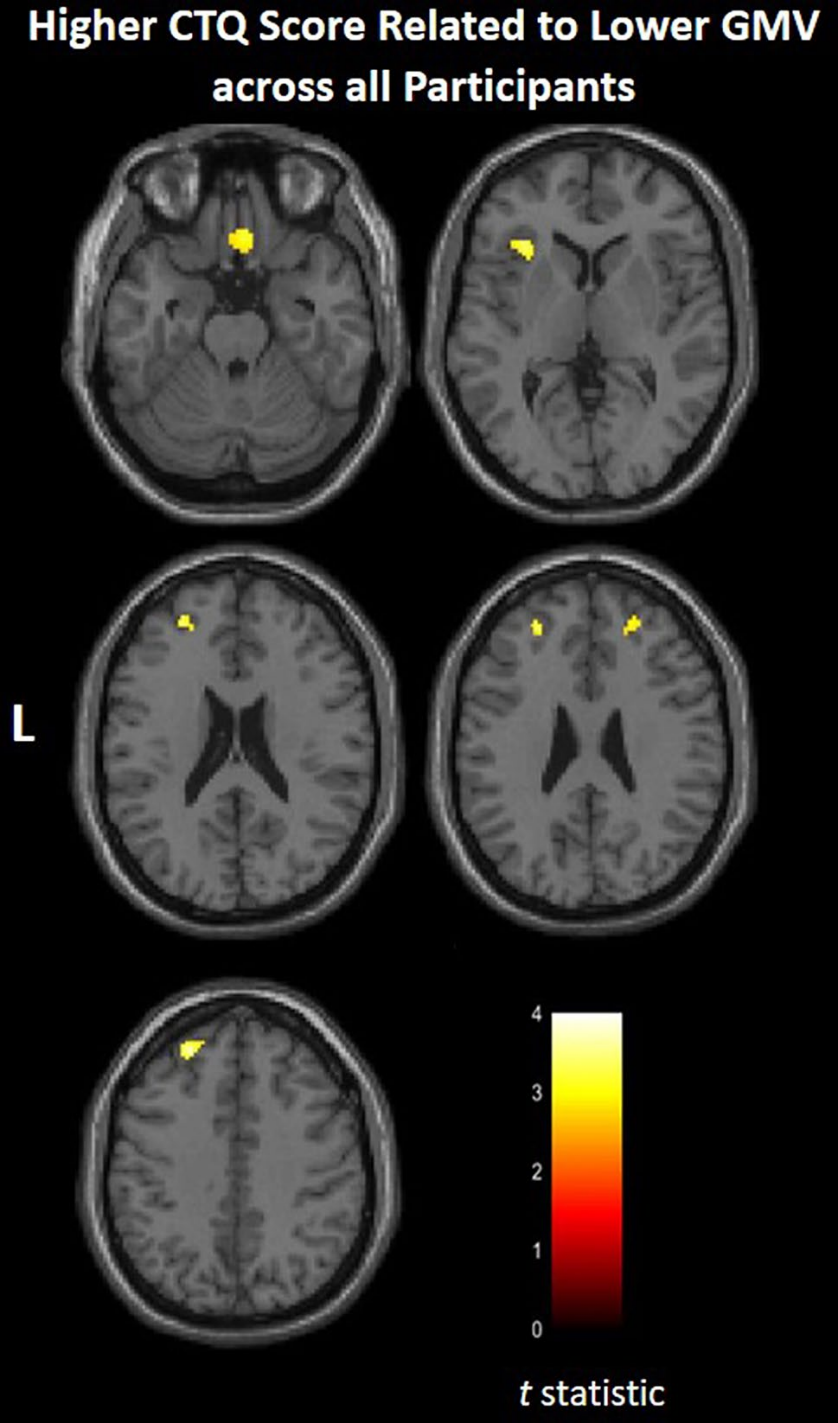
Areas of gray matter volume that related to childhood maltreatment. Axial-oblique images show regions where gray matter volume (GMV) inversely correlated with Childhood Trauma Questionnaire (CTQ) total scores across all participants [total N = 47; participants without a first-degree family member diagnosed with bipolar disorder (FH−): N = 26; participants with first-degree family member(s) diagnosed with bipolar disorder (FH+): N = 21]. Significance threshold is p < 0.005, uncorrected; clusters > 20 voxels. Left of figure denotes left side of brain. Color bar represents range of T values.

As shown in Fig. 1, greater total CTQ scores correlated with lower GMV in two clusters in ventral prefrontal cortex (Brodmann Area [BA] 11, Montreal Neurological Institute [MNI] space coordinates: x = 3 mm, y = 27 mm, z = − 24 mm, cluster = 141 voxels; BA47, x = 39 mm, y = 27 mm, z = − 14 mm, cluster = 53 voxels), a cluster in left insula (x = − 32 mm, y = 24 mm, z = 5 mm, cluster = 150 voxels), a clusters in left rostral prefrontal cortex (BA10, x = − 26 mm, y = 47 mm, z = 24 mm, cluster = 114 voxels), and two clusters in bilateral dorsolateral prefrontal cortex (BA9, x = 26 mm, y = 47 mm, z = 29 mm, cluster = 203 voxels; x = − 23 mm, y = 45 mm, z = 42 mm, cluster = 754 voxels) across all young adults. No significant group by CTQ score interactions on GMV in prefrontal-paralimbic ROIs were observed when comparing FH+ and FH− groups.

#### Childhood maltreatment and associated prefrontal-paralimbic gray matter volume: relations with alcohol/cannabis use patterns

There was a family history group (FH+ vs. FH−) by insula GMV interaction on average number of drinks consumed per drinking day over the past four weeks (p = 0.03). Stratifying by group revealed a negative relation between insula GMV and average number of drinks per drinking day in the FH+ group (p = 0.02), but not in the FH− group (p = 0.7). See Table 3 for model statistics. Additionally, when examining relations between CTQ and associated GMV with alcohol use across all participants, age of alcohol initiation was inversely related to average number of drinks per drinking day and maximum number of drinking days per week (p’s = 0.01). Across all individuals who reported past month cannabis use, lower insula GMV significantly correlated with greater maximum number of cannabis use days per week. CTQ was not related to recent alcohol or cannabis use when looking across all participants, and there was no CTQ by group interaction on substance use.

**Table 3:** Insula gray matter volume in relation to alcohol/cannabis use patterns.

	t Ratio	SE	p value
**Insula gray matter volume (GMV): average drinks/drinking day**			
Family history group by insula GMV interaction on average drinks/drinking day	− 2.3	1.2	**0.03**
Model summary	F_(5,41)_ = 2.9, p = **0.02**, R^2^ = 0.3		
FH+: average drinks/drinking day	2.8	1.3	**0.02**
Model summary	F_(3,16)_ = 10.9, p = **0.0007**, R^2^ = 0.7		
FH−: average drinks/drinking day	0.4	2.1	0.7
Model summary	F_(3,24)_ = 0.3, p = 0.8, R^2^ = 0.04		
**Insula gray matter volume (GMV): maximum number of cannabis use days/weeks**			
Maximum number of cannabis use days/week	− 3.3	0.8	**0.01**
Model summary	F_(3,12)_ = 5.1, p = **0.02**, R^2^ = 0.6		

Relations between insula gray matter volume (GMV) with alcohol and cannabis use measures. Analyses with alcohol measures as the dependent variable were performed across all participants (bipolar disorder family history negative (FH−): N = 25; bipolar disorder family history positive (FH+): N = 17) who completed the Timeline Follow Back and reported lifetime alcohol use, using GMV extracted from clusters showing a significant negative relationship with Childhood Trauma Questionnaire (CTQ) total score. Analysis with the cannabis measure as the dependent variable was performed only in past month cannabis user (FH−: N = 6; FH+: N = 7). Results are reported for the overall models, the family history group by insula GMV interaction, and the alcohol/cannabis use factors within each model. SE = standard error.

#### Exploratory analysis: FH+ subgroups

**Figure 2:**
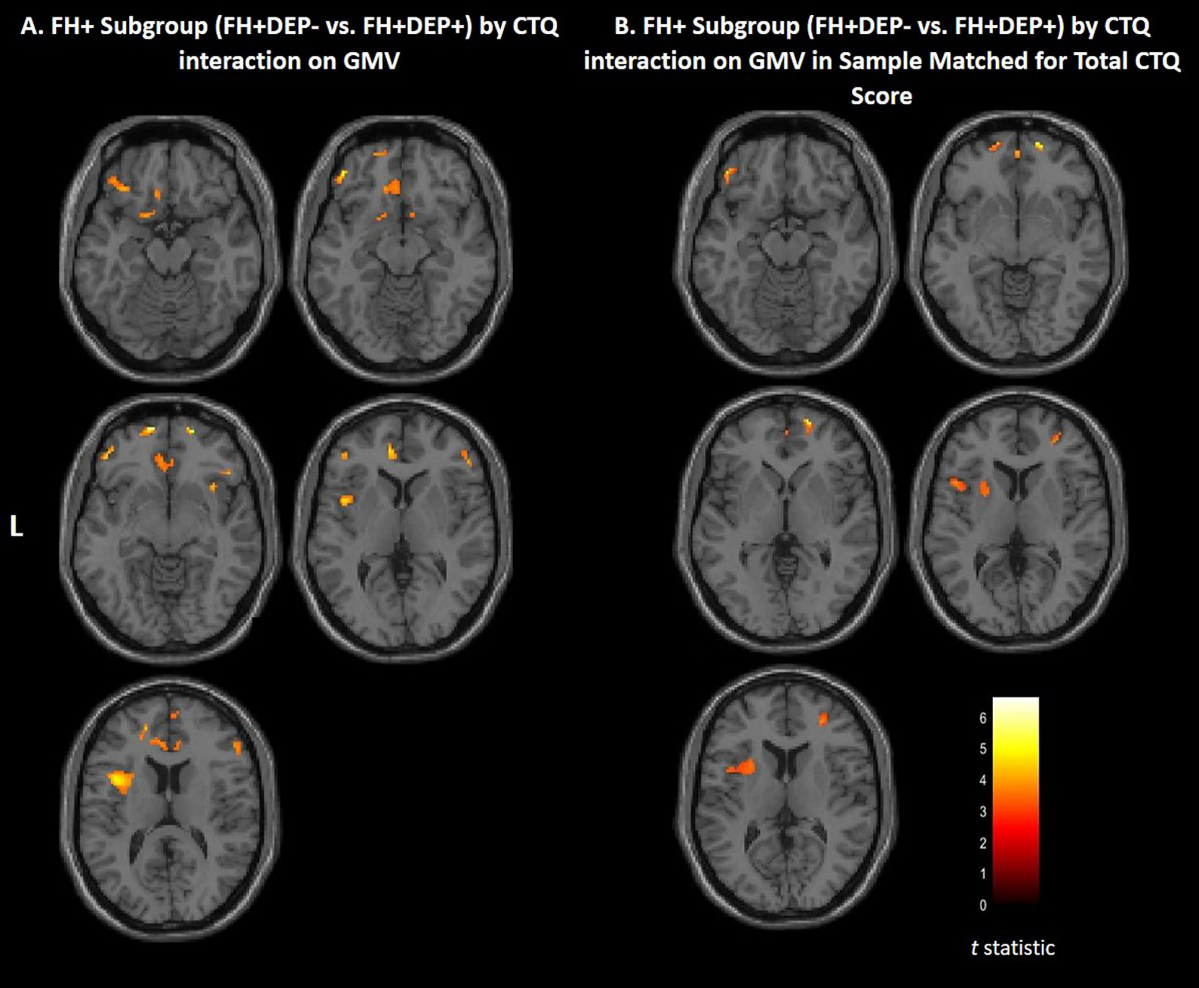
Areas of gray matter volume that showed childhood maltreatment by FH+ subgroups (FH+DEP−, FH+DEP+) interaction. (**A**) Axial-oblique images show regional gray matter volume (GMV) that showed a significant family history positive (FH+) subgroup by Childhood Trauma Questionnaire (CTQ) total score interaction. Higher CTQ score correlated with lower GMV in FH+ individuals with a history of unipolar depression (FH+DEP+), and conversely, greater GMV, or no significant relation, in these clusters in FH+ individuals without a history of unipolar depression (FH+DEP−). Total N = 21; FH+DEP−: N = 9; FH+DEP+: N = 12. (**B**) Axial-oblique images show regional GMV that showed a significant bipolar disorder FH+ subgroup by CTQ total score interaction when removing the four individuals with highest CTQ scores in the FH+DEP+ subgroup so groups were matched on total CTQ scores. Higher CTQ score was related to lower GMV in FH+DEP+ individuals, and conversely, greater GMV, or no significant relation, in these clusters in FH+DEP− individuals. Total N = 17; FH+DEP−: N = 9; FH+DEP+: N = 8). Significance threshold is p < 0.005, uncorrected; clusters > 20 voxels. Left of figure denotes left side of brain. Color bar represents range of T values.

CTQ scores were higher in the FH+DEP+ group compared to the FH+DEP− group (p = 0.01) (Table 1). FH+ subgroups did not differ in recent alcohol or cannabis use (Table. 2). Within the FH+ group, there was a significant subgroup (FH+DEP+, FH+DEP−) by CTQ interaction on GMV in the ventral extending to rostral and dorsal prefrontal, and insular cortex (Fig. 2A). See Table 4 for cluster details and post hoc modeling results. Post hoc analyses revealed that higher CTQ scores correlated with lower GMV in the FH+DEP+ group, and conversely associated with greater GMV, or no significant correlation, in the FH+DEP− group. Modeling the interaction after removing the four FH+DEP+ individuals with highest CTQ scores revealed a similar group by CTQ interaction on GMV in the ventral extending to rostral and dorsal prefrontal, and insular cortex. Figure 2B shows significant FH+ subgroup (FH+DEP+, FH+DEP−) by CTQ interaction after removing four individuals with the highest CTQ score in the FH+DEP+ subgroup.

**Table 4:** Areas of gray matter volume showing a significant FH+ subgroup (FH+DEP− vs. FH+DEP+) by Childhood Trauma Questionnaire total score interaction.

	Cluster localization and MNI coordinates at cluster peak	FH+DEP− (N = 9)			FH+DEP+(N = 12)		
		t Ratio	SE	p-value	t Ratio	SE	p-value
Ventral prefrontal cortex	Left Brodmann Area 11 x = − 18 mm, y = 23 mm, z = − 21 mm, clusters = 21 voxels	3.16	0.002	0.02	− 3.45	0.0005	0.007
		F_(2,8)_ = 5.0, p = 0.05, R^2^ = 0.6			F_(2,11)_ = 13.0, p = 0.002, R^2^ = 0.7		
	Left Brodmann Area 11 x = − 15 mm, y = 11 mm, z = − 15 mm, clusters = 81 voxels	2.9	0.001	**0.03**	− 3.6	0.0005	**0.006**
		F_(2,8)_ = 4.8, p = 0.06, R^2^ = 0.6			F_(2,11)_ = 7.2, p = **0.01**, R^2^ = 0.6		
	Left Brodmann Area 47 x = − 44 mm, y = 42 mm, z = − 14 mm, clusters = 295 voxels	3.9	0.001	**0.008**	− 5.0	0.0005	**0.008**
		F_(2,8)_ = 8.4, p = **0.02**, R^2^ = 0.7			F_(2,11)_ = 12.4, p = **0.003**, R^2^ = 0.7		
	Right Brodmann Area 11 x = 5 mm, y = 50 mm, z = − 18 mm, clusters = 26 voxels	2.2	0.001	0.07	− 4.9	0.0005	**0.0008**
		F_(2,8)_ = 3.5, p = 0.1, R^2^ = 0.5			F_(2,11)_ = 12.3, p = **0.003**, R^2^ = 0.7		
	Right Brodmann Area 11 x = 9 mm, y = 12 mm, z = − 14 mm, clusters = 23 voxels	1.8	0.001	0.12	− 4.6	0.0006	**0.001**
		F_(2,8)_ = 1.6, p = 0.3, R^2^ = 0.3			F_(2,11)_ = 11.3, p = **0.004**, R^2^ = 0.7		
	Right Brodmann Area 47 x = 41 mm, y = 27 mm, z = − 5 mm, clusters = 67 voxels	2.4	0.001	**0.05**	− 5.5	0.0007	**0.0004**
		F_(2,8)_ = 2.9, p = 0.1, R^2^ = 0.5			F_(2,11)_ = 15.**6**, p = **0.001**, R^2^ = 0.8		
Rostral and dorsal prefrontal cortex	Left Brodmann Area 10 x = − 12 mm, y = 62 mm, z = − 8 mm, clusters = 110 voxels	5.11	0.0006	**0.002**	− 5.7	0.0004	**0.0003**
		F_(2,8)_ = 18.2, p = **0.003**, R^2^ = 0.9			F_(2,11)_ = 18.2, p = **0.0007**, R^2^ = 0.8		
	Left Brodmann Area 10 x = − 17 mm, y = 48 mm, z = 14 mm, clusters = 34 voxels	3.4	0.001	**0.02**	− 2.4	0.0004	**0.04**
		F_(2,8)_ = 5.7, p = **0.04**, R^2^ = 0.7			F_(2,11)_ = 4.7, p = **0.04**, R^2^ = 0.5		
	Right Brodmann Area 10 x = 39 mm, y = 47 mm, z = − 12 mm, clusters = 20 voxels	2.9	0.001	**0.03**	− 3.2	0.0007	**0.01**
		F_(2,8)_ = 4.1, p = 0.08, R^2^ = 0.6			F_(2,11)_ = 5.2, p = **0.03**, R^2^ = 0.5		
	Right Brodmann Area 46 x = 50 mm, y = 38 mm, z = 9 mm, clusters = 444 voxels	2.9	0.002	**0.03**	− 4.8	0.0007	**0.001**
		F_(2,8)_ = 5.4, p = **0.05**, R^2^ = 0.6			F_(2,11)_ = 12.0, p = **0.003**, R^2^ = 0.7		
	Right Brodmann Area 10 x = 9 mm, y = 60 mm, z = 17 mm, clusters = 70 voxels	2.1	0.002	0.09	− 4.4	0.0005	**0.002**
		F_(2,8)_ = 2.8, p = 0.1, R^2^ = 0.5			F_(2,11)_ = 10.7, p = **0.004**, R^2^ = 0.7		
Insula	Left Insula x = − 44 mm, y = 6 mm, z = 8 mm, clusters = 864 voxels	3.1	0.002	**0.02**	− 3.9	0.0008	**0.003**
		F_(2,8)_ = 4.7, p = 0.06, R^2^ = 0.6			F_(2,11)_ = 7.8, p = **0.01**, R^2^ = 0.6		
	Right Insula x = 36 mm, y = 17 mm, z = − 6 mm, clusters = 77 voxels	2.7	0.001	**0.04**	− 4.0	0.0008	**0.003**
		F_(2,8)_ = 3.6, p = 0.09, R^2^ = 0.5			F_(2,11)_ = 8.8, p = **0.009**, R^2^ = 0.6		

Brodmann Area and Montreal Neurological Institute space coordinates for clusters showing a significant FH+ subgroup by Childhood Trauma Questionnaire (CTQ) total score interaction. Using extracted gray matter volume (GMV) from these clusters, relations between CTQ total score and extracted GMV were modeled stratified by bipolar disorder family history positive (FH+) subgroups [FH+ individuals without a history of unipolar depression (FH+DEP−); FH+ individuals with a history of unipolar depression (FH+DEP+)] while covarying biological sex. Results are reported for the overall model for each cluster, and the GMV factor within each model for each cluster.

## Discussion

Results from this preliminary study suggest a history of childhood maltreatment is related to lower prefrontal ventral, rostral, and dorsal prefrontal, and insular cortical GMV in young adults, as previously reported^6^. We predicted familial risk for bipolar disorder, and hence presumably genetic vulnerability, would be associated with greater magnitude of GMV differences. Although this prediction was not supported, in an exploratory analysis we did observe a significant interaction between familial risk for bipolar disorder subgroups (i.e. those already expressing depressive symptoms versus those not) and childhood maltreatment on GMV. The additional requirement of the presence of mood symptoms may refine the FH+ group to one enriched for higher genetic risk^20^, because the majority of individuals with parental risk for bipolar disorder do not develop illness^41^, and presumably do not inherit the necessary genetic risk profile. Additionally, lower insula GMV was associated with greater number of average drinks consumed per drinking day, with this relationship only observed in individuals with familial risk for bipolar disorder. Lower insula GMV was also associated with greater frequency of cannabis use across all participants. Although preliminary, these findings could suggest that greater maltreatment-related structural changes—previously observed in bipolar disorder^7–9^—may emerge over time, following alcohol/cannabis misuse, and be related to genetic vulnerability for bipolar disorder. This preliminary study supports further research on the interactions between childhood maltreatment, family history (focusing on genetically enriched subgroups), and substance use on long-term neural and clinical outcomes.

The prefrontal cortex and insula are highly sensitive to the effects of stress^42,43^, with childhood and adolescence marking a period of increased vulnerability to environmentally triggered insults due to the prolonged maturation of these regions during this developmental epoch^44,45^. In line with our findings, previous studies have consistently shown childhood maltreatment-related reductions in the prefrontal cortex, especially within the ventral and dorsolateral prefrontal cortex, and insula GMV in typically developing and psychiatric populations^6,34,46^. The prefrontal cortex, both ventral and dorsal components, and the insula contribute to emotional regulation, impulse control, and risky decision-making^47,48^, with disruption in these processes thought to contribute to risk for the development of SUDs^49^. Whereas this cross-sectional study cannot specify who will develop alcohol/cannabis use problems, a recent longitudinal study found that lower prefrontal, including ventral, rostral and dorsolateral prefrontal cortex, and insula GMV distinguished individuals with bipolar disorder who subsequently developed alcohol/cannabis use problems^18^. These findings are concordant with our preliminary results—suggesting variation in insula GMV, particularly in individuals with familial risk for bipolar disorder, may be associated with greater alcohol/cannabis use—and support that structural changes within these regions following ELS may increase risk for alcohol/cannabis use problems. Additionally, alcohol use, even when consumed at moderate levels, has been associated with greater bipolar illness severity^50^. Therefore, understanding how brain structure relates to moderate alcohol use (and vice versa) in individuals at risk for bipolar disorder may improve our understanding of mechanisms that contribute to mood symptom onset and progression. Our results further suggest that familial factors may interact with childhood maltreatment-associated insular structural differences to increase alcohol use. It is possible that FH+ individuals possess additional brain differences (e.g. white matter) not elucidated in the current study that decrease these individual’s ability to compensate for GMV deficits^51^. Lower insula GMV was also associated with greater frequency of cannabis use over the past month. Unfortunately, the small sample size of individuals who used cannabis prevented us from investigating group by GMV interactions on cannabis use patterns. Not only has recent work found the insula to play a key role in the development of addiction through its involvement in drug-craving and -seeking, but studies have also suggested insula abnormalities may stem from genetic, environmental, or developmental factors (or their combination) that precede onset of problematic substance use^52^.

Interestingly, childhood maltreatment was not directly related to alcohol/cannabis use. While we cannot infer causality from this study, it is possible early life stress is indirectly associated with greater alcohol use, especially in those with greater genetic risk for bipolar disorder, and greater maltreatment-related differences in the brain—previously reported in bipolar disorder^7–9^—may emerge over time. Additionally, a more enriched genetic risk group (i.e., presence of prodromal symptoms or identified through genetic testing) may reveal more robust results. Further supporting this are prior studies suggesting unipolar depression family history by ELS interactions on neural phenotypes^14^ and gene by ELS interactions on risk for, and symptom severity in, bipolar disorder^53^. Interestingly, the low risk FH+ subgroup (FH+DEP−) exhibited a positive relationship between childhood maltreatment and several GMV clusters in the ventral, rostral, and dorsal prefrontal cortex. While speculative, increased GMV in FH+DEP− individuals may be neuroprotective. In fact, work has suggested neuroprotective mechanisms may prevent development of illness in asymptomatic individuals with bipolar familial risk^54,55^. Additionally, recent longitudinal work has indicated neural markers of risk and resilience in individuals with familial risk for bipolar disorder^56^. Future longitudinal work examining interactions between childhood maltreatment, familial risk, and substance use, and how associated brain changes relate to development of problematic substance use and neural trajectories, is critically needed to understand mechanisms and familial factors that confer risk for psychopathology.

### Limitations

Findings from this preliminary cross-sectional study should be interpreted with caution due to a relatively small sample size and heterogeneous FH+ group characteristics. Causation cannot be determined without within subject experimental designs that directly link neuroanatomical differences to outcomes over time. Likewise, while GMV changes may contribute to risky alcohol use patterns in those with familial risk for bipolar disorder, it is also possible that individuals with familial risk may be more vulnerable to the negative consequences of alcohol use which in turn may contribute to brain differences. This study also did not investigate age of childhood maltreatment onset or duration. Differences in ELS severity, onset, and duration could have contributed to results^57^, and future studies should incorporate these maltreatment factors.

We recruited a generalizable comparison group, which included some with family history of psychopathology other than bipolar disorder, but the FH− group had lower CTQ total scores compared to the FH+ group. The FH+ group also had a greater prevalence of family history of anxiety disorders, compared to the FH− group; hence results may not be specific only to familial risk for bipolar disorder. However, the two FH+ subgroups (stratified by depression history) did not significantly differ on these familial factors and we were able to match CTQ scores for a sensitivity analysis following a significant subgroup by CTQ interaction on GMV. Neuroimaging results were considered significant at p < 0.005 uncorrected in hypothesized regions, and we did not correct for multiple comparisons when looking at childhood maltreatment and GMV relations with a priori substance use variables. These thresholds were set to avoid strict correction and to minimize type II errors and generate hypotheses for future studies, as previously suggested^18,35^. However, by not using a more conservative correction method there is greater risk for type I errors. Findings should therefore be considered hypothesis generating and interpreted with caution. Future longitudinal studies with larger sample sizes, more comprehensive assessment of maltreatment—including onset and duration—and more homogeneous familial risk groups, are needed to confirm and extend findings. Additionally, this work would benefit from examining individuals’ genetic loading and incorporating other emerging genetic methods, including investigation of polygenic risk scores and epigenetic modifications.

## Conclusion

Our findings are concordant with the current literature suggesting that childhood maltreatment is inversely related to prefrontal-paralimbic GMV. While preliminary, results suggest familial factors may relate to alcohol use patterns following childhood maltreatment. Not all individuals who exhibit childhood maltreatment-related GMV abnormalities develop psychopathology, and more research on factors that contribute to different clinical outcomes following maltreatment is critically needed for early intervention and prevention strategies.

## Supplementary Information

Supplementary Information.
