# Supplementary material for: Childhood maltreatment, prefrontal-paralimbic gray matter volume, and substance use in young adults and interactions with risk for bipolar disorder
Source: Sci Rep. 2021 Jan 8;11:123. doi: 10.1038/s41598-020-80407-w (PMC7794246; doi:10.1038/s41598-020-80407-w)
Supplement: Supplementary file 1 — Supplementary Information. [file 41598_2020_80407_MOESM1_ESM.docx]

**Childhood Maltreatment, Prefrontal-Paralimbic Gray Matter Volume, and Substance Use in Young Adults and Interactions with Risk for Bipolar Disorder**

**Dylan E Kirsch^1,2,3^, Valeria Tretyak^1,2,4^, Sepeadeh Radpour^1^, Wade A Weber^1^, Charles B Nemeroff^1,2,5^, Kim Fromme^2,4^, Stephen M Strakowski^1,2,3,4^, and Elizabeth TC Lippard*^1,2,3,4,5^**

**Supplemental Table 1: Demographic, childhood maltreatment, and familial factors stratified by FH+ subgroup (FH+DEP- vs. FH+DEP+) in sample matched for average Childhood Trauma Questionnaire score**

Bipolar family history positive (FH+) subgroup between-group [FH+ individuals without a history of unipolar depression (FH+DEP) vs. FH+ individuals with a history of unipolar depression (FH+DEP+)] differences when removing four FH+ individuals with highest Childhood Trauma Questionnaire (CTQ) scores. Differences in age, IQ, and CTQ, were compared using a two-sample t-test. All other factors were examined with a Mann-Whitney Wilcoxon Test, Chi-square, or Fisher Exact tests, as appropriate. ^U^ represents p-values calculated with Mann-Whitney Wilcoxon Test. ^F^ represents p-values calculated with Fisher exact test. ^1^FSIQ-2 represents the composite score for the full-scale intelligence quotient comprising verbal comprehension and matrix reasoning subtests on the Wechsler Abbreviated Scale of Intelligence-Second Edition (WASI-II). ^2^Childhood Trauma Questionnaire. ^3^Anxiety disorders included generalized anxiety disorder, specific phobia, panic disorder, and social anxiety disorder.

| **Supplemental Table 1** |
| --- |
| Demographics |
| CTQ^2^ |
| Cormorbidities |
| Urinalysis Toxicology Screen |
| Family  History |

|  | **FH+**  **DEP- (N=9)** | **FH+**  **DEP+**  **(N=8)** | **p value** |
| --- | --- | --- | --- |
| Mean Age (SD) | 21 (2) | 21 (2) | 0.9 |
| Number of Females (%) | 6 (67) | 7 (88) | 0.6^F^ |
| Mean WASI-II FSIQ-2^1^ (SD) | 117 (14) | 116 (9) | 0.9 |
| CTQ Total Score (SD) | 36 (3) | 42 (3) | 0.16 |
| CTQ Range | 27-53 | 26-59 |  |
| Current Alcohol Use Disorders (AUDs): |  |  |  |
| AUD - mild (%) | 0 | 0 | — |
| Past Alcohol Use Disorders (AUDs): |  |  |  |
| AUD - mild (%) | 1 (11) | 0 | 0.4 ^F^ |
| Current Substance Use Disorder (SUDs): |  |  |  |
| Cannabis Use Disorder - mild (%) | 1 (11) | 0 | 0.4 ^F^ |
| Cannabis Use Disorder - moderate (%) | 0 | 0 | — |
| Past Substance Use Disorder (SUDs): |  |  |  |
| Cannabis Use Disorder - mild (%) | 0 | 0 | — |
| Cannabis Use Disorder - severe (%) | 0 | 1 (13) | 1^F^ |
| Past Major Depressive Episode (%) | 0 | 8 (100) | **<0.001 ^F^** |
| Anxiety Disorders^3^ (%) | 2 (22) | 2 (25) | 1^F^ |
| Tetrahydrocannabinol (%) | 1 (11) | 0 | 1 ^F^ |
| Amphetamines (%) | 0 | 0 | — |
| Bipolar Disorder (%) | 9 (100) | 8 (100) | 1 ^F^ |
| Depression (%) | 4 (44) | 4 (50) | 1 ^F^ |
| Anxiety (%) | 5 (56) | 5 (63) | 1^F^ |
| ADHD (%) | 1 (11) | 2 (25) | 0.6 ^F^ |
| Alcohol Use Problems (%) | 7 (78) | 7 (88) | 1^F^ |
| Substance Use Problems (%) | 4 (44) | 5 (63) | 0.3 ^F^ |
